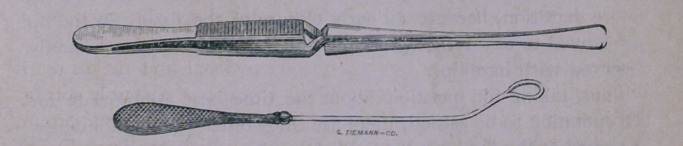# On a Few Instruments for the Practical Treatment of Uterine Diseases

**Published:** 1871-02

**Authors:** Philip Adolphus

**Affiliations:** Chicago


Article IV.—*On a few Instruments for the Practical Treatment of Uterine Diseases.* By Philip Adolphus, M.D., Chicago.

The object of the writer in these articles is to describe the instruments which he has found most efficient in the diagnosis and treatment of Uterine Diseases, after repeated trials of a great variety not only in his private clientèle but also in a somewhat extensive dispensary practice.

### NOTT'S SPECULUM.

The fact that the show cases of the instrument dealer groan with different kinds of Specula, and that almost every journal brings us a description of another modification, should be an evidence to us, that the instruments we possess are not perfect. Scanzoni says: * "We have experimented with instruments of the most diverse construction, and we are convinced that none of those now known completely answer the purpose."

All the cylindrical and valvular specula now in use have for their object the verification by the eye of the diagnosis made by the touch, of the various stages of disease of the *vagina and cervix uteri*, and the color and consistence of the accompanying discharges; and furthermore, the application of instruments and remedial agents to these parts.

* Diseases of Sexual Organs of Women. Fol. 44.

The length of the blades of these instruments and their immobility after insertion, interfere with treatment, for, as Lisfranc observes,* "In cases requiring operations about the cervix, all that the surgeon has to do is to lay hold of the os uteri with a hook, and draw it gently down until it comes fairly within sight." This is a very serious objection, for the hook or its substitute, a tiny vulsellum, is now advantageously used for the purpose of steadying the womb and drawing it downwards in every operation, great or small. In the introduction of the probe for the purposes of diagnosis, in the constant probings instituted whilst handling instruments, for the purpose of guiding them safely, painlessly and efficiently to their destination; in applications to the cervix and fundus, in inserting sponge tents and removing them, in intra-uterine scarifications and in the introduction of the uterine catheter for the purpose of cleaning the organ of its morbid secretions; in all these cases we absolutely need the hook or vulsellum to draw down and then steady the uterus.

It is almost impossible to probe the cavity of the womb with valvular and cylindrical specula in situ; it consequently follows that no instrument can be easily introduced into that organ through these specula. The immense loss to *diagnosis* and especially to *treatment* will become apparent when the remarks on the Uterine Sound are perused. Generally speaking, the diagnosis regarding the size of the uterus, its versions, flexions, consistence and mobility, with any existing complications, can be ascertained by the vaginal and rectal touch, combined with abdominal palpation. Not always, however, can this success be attained by manipulators. The experienced skill of a Sims "may determine the condition of the uterus as easily as we would that of a pear in a napkin," yet there are very many cases, where fright, pain, nervousness, natural stupidity, excessive adipose deposit of the walls of the abdomen, tympanitic distention, inflammation of parts and spasmodic contraction of the abdominal parietes, prevent the attainment of even an approximation to a correct idea of the state and position of the uterus on the first examination by the touch. Nay, repeated examinations are necessary under these circumstances where anæsthetics cannot be used.

* Sir J. Y. Simpson, Obstetric Memoirs, 1855. Note, fol. 61.

In these cases, and they occur frequently, a proper speculum will permit us to introduce the probe gently, easily, successfully; and gather all the information which this instrument *thus employed* is capable of giving.

Finally, the valvular specula dilate the vagina by mechanical force, often produce much pain by distention and pinching of parts, hide from view diseased conditions of the vagina, and are useless in all operations where the knife and scissors are necessary. In short, the limits of their usefulness consist in permitting an ocular examination of the cervix and the treatment of its maladies.

It cannot be controverted that the *beau ideal* of a good speculum is that of Sims. To enumerate its qualities and capacities is to recapitulate every necessary requisite for the easy performance of the simplest as well as the most difficult of manipulations. Every speculum claiming superior advantages will be gauged by its approximation to the good qualities of that of Sims. His merit in the introduction of this great boon to Gynecology consists in the fact that he took advantage of an accident, which happening to an ordinary man, would have borne no fruit to humanity.

I cannot do better than to quote the words of Thomas in this connection.* "I have elsewhere called the labors of Récamier and Simpson eras in the progress of this department. I now venture so to style those of Marion Sims. In doing this I make no reference to the improvements inaugurated by him in the treatment of injuries to the genital organs; my allusion is to the great advantages which now flow and are to flow from the invention of his speculum, which exposes the uterus by a new principle and opens the way to a more complete examination of this organ.

"Récamier marked an era by improving our powers of diagnosis in exposing the cervix uteri; Simpson, another, by opening to investigation the body of the uterus; and Sims, a third, by rendering both investigations more simple, complete and satisfactory. The ordinary specula in use before the discovery of Sims' simply separate the walls mechanically, and thus expose the uterus. Sims' instrument, on the other hand, elevates the posterior vaginal walls, which allows the entrance of air to distend the whole passage, the woman lying on her side in such a manner that the cavity can be probed with the most perfect ease, and applications

* Diseases of Women, by T. G. Thomas. 1869 Fol 49.

made to the fundus. I am fully aware that many will differ with me in opinion, but being entirely free from prejudice in favor of this instrument, or against the ordinary varieties, I maintain it fearlessly, feeling confident that time will prove it to be correct. No one who has not tested the two methods of examination is really entitled to an opinion upon the point, and I cannot doubt the conclusion of him who has done so faithfully and intelligently."

Although the writer of this article is delighted to bear testimony that the little he knows of practical manipulations has been acquired by the use of Sims' Speculum and Probe; still he must admit that whilst some objections advanced by authors against the use of this speculum have no weight, there are others, which do not make it expedient to introduce it in the ordinary operations performed in private practice.

According to authors, a skilled assistant cannot be dispensed with, a table, a chair or bed three feet high is necessary, a horizontal light is requisite.

It must be admitted that in cutting operations a skilled assistant is requisite; it would be desirable also in ordinary operations, but it is not necessary. The correct position of the patient is indispensable; for the rest, a light and steady hand to retain the speculum is the requisite qualification of the assistant. Over fifteen hundred examinations have thus been made by the writer, with the assistance of such of his dispensary patients as happened for the time being to be present for treatment.

The chief objection against its use, however, *is the presence of a third party* when that presence is not desired.* "The amount of confidence shown to the profession by women in this respect, varies extremely, but I may safely say, that it is greatest in proportion to their rank and mental culture, for while women of the lower and middling classes have not the delicate perception of implicit trust, those of the higher feel that they can rely on the honor of gentlemen, and are generally of opinion *that it is sufficiently painful to submit to an examination without having the additional annoyance of its being witnessed even by another.* The best plan, therefore, is to let patients do just as they

* Uterine Therapeutics, E. J. Tilt, M.D. 1869 Fol. 11.

like, without objecting to, or requiring, the presence of a third party."

To Dr. John C. Nott, of New York City,* belongs the distinguished honor of having introduced to the profession a speculum, which, when it becomes generally known will be in the hands of every practitioner at home or abroad. Its qualities are such that it must supercede all specula (excepting Sims') now known. Another era has dawned on Gynecology, for by its use any instrument can be introduced into the uterus with ease and safety, and the patient may be placed on a bed or couch in the usual position (on the back), the practitioner requires no assistants, the instrument, easily introduced, is self-retaining, does not pinch or stretch the parts, and produces no pain on introduction. The uterus can be drawn towards the operator, whilst the speculum is in situ, the parts will then be felt as well as seen, and any operation performed which the practitioner would desire to perform without an assistant.

No one can form an adequate idea of this instrument without having seen and tried it. It is correct in design and simple in its construction. In the accompanying figure it appears formidable and complicated. I shall copy the author's description of his instrument, and add such comments (in brackets) as occur to me.

[This tri-valve instrument consists of a large duck-bill four inches long, to which are attached two small valves or feet of three inches in length; these are fastened by means of chains, to a screw which opens or closes the instrument.]

* Vide American Journal Med. Sciences, October, 1868, fol. 420, and American Journal of Obstetrics, Nov. 1869, fol. 490.

"To the large blade, or duck-bill, is attached a loop of strong steel wire, which may be slided forward or backward, according to the depth of the vagina. This acts as a depressor on the posterior wall of the vagina, but is rarely required except in large fat women."

"The two feet or small blades, which rest on the rami of the pubes when the instrument is introduced, are attached to a screw by means of chains. At the extremities of the feet, are fixed on the inner surface small steel plates, which may be pushed out to increase its length."

"These two feet are so shaped as to curve smoothly around the bones, and not to press on their sharp edges." [Their length is three inches, their width one-half inch, when fully expanded the separation of these little blades at the ostium vagina is two inches, whilst at the terminus of the small blades the distance is three and one-half inches. The vertical distance from either of the small blades to the bottom of the duck-bill, when fully expanded, is, at the ostium vagina, two inches, and at the end of the little blades, three inches. Let any one examine the speculum so disended, and he will at once perceive that Mr. F. A. Stohlman, of the firm of G. Tieman & Co., New York, has executed Nott's design with the ingenuity of a master hand.]

"The instrument is expanded by means of a button which plays up and down the screw, expands and contracts all the blades simultaneously, and fixes them at any point to suit the capacity of the vagina. Thus expanded, the anterior wall of the vagina is pushed out of the way [by a Sims' Depressor] and the os uteri brought into view, a single tenaculum [Sims'] or a double spring tenaculum [Nott's] is then fixed in the anterior lip, and the organ is drawn forward, placed and held in the position we desire."

I have removed the loop of strong steel wire, which is attached to the large blade, as it is seldom required, and can be introduced whilst the instrument is in situ, should a very long vagina make it necessary.

"The small steel plates which are fixed at the inner surface of the extremities of the feet, which may be pushed out to increase its length," I have never used, and cannot conceive, when, why, and for what purpose they should be used, and I would respectfully suggest to the inventor, that he would request Mr. Stohlman not to add them in future. I know that the instrument would become more valuable without them.

To prevent pain to the patient and difficulty to the operator, whilst the instrument is expanding, the screw should be always oiled.

Thus used, the instrument is destined to make the tour of the globe, to confer blessings on women, and to advance the science of Gynecology.

Fifty-four years have now elapsed since Récamier introduced his Speculum; twenty-eight years have rolled away since the illustrious Simpson opened the interior of the uterus to manipulations; again, have twenty years passed since another great man by the invention of the speculum insured ease and safety to investigations, where previously the greatest skill was only rewarded by riskful uncertainty; and now J. C. Nott gives us an instrument which permits the general practitioner with a little practice to emulate the skill of the giants of our profession.

*We, nevertheless, continue to use Récamier's or its modifications at this day almost exclusively.*

NOTE. Before me lies a duck-bill of English make (Meadows' patented, Meyer & Melzer, London), identical in principle with Sims' and Nott's. It is a trivalve, but inferior to Nott's. The duck-bill is too long, $4\frac{3}{4}$ inches; the upper blades are too long, 4 inches, and too straight. It is nevertheless an excellent instrument.

Sims' Depressor, used for the purpose of placing the os uteri within sight of Sims' Hook, and Sims' Hook for grasping the lip of the os, and drawing it in the position required, are well known. I, however, prefer Nott's Vulsellum, for it has the advantages of holding the lip of the womb firmly without slipping, even when the hold of the operator on the instrument is relaxed. Neither of these instruments give pain.

All these instruments may be procured of Messrs. Bliss & Sharp, 144 Lake street, Chicago, the agent of Messrs. Tieman & Co., of New York City.

## Figures and Tables

**Figure f1:**
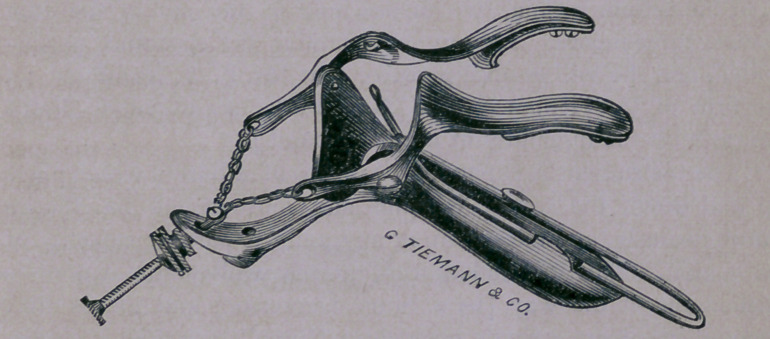


**Figure f2:**